# Why Did the Meerkat Cross the Road? Flexible Adaptation of Phylogenetically-Old Behavioural Strategies to Modern-Day Threats

**DOI:** 10.1371/journal.pone.0052834

**Published:** 2013-02-18

**Authors:** Nicolas Perony, Simon W. Townsend

**Affiliations:** 1 Chair of Systems Design, ETH Zurich, Zurich, Switzerland; 2 Institute of Evolutionary Biology and Environmental Studies, University of Zurich, Zurich, Switzerland; 3 Kalahari Meerkat Project, Kuruman River Reserve, Northern Cape, South Africa; University of Bristol, United Kingdom

## Abstract

Risk-sensitive adaptive spatial organisation during group movement has been shown to efficiently minimise the risks associated with external ecological threats. Whether animals can draw on such behaviours when confronted with man-made threats is generally less clear. We studied road-crossing in a wild, but habituated, population of meerkats living in the Kalahari Desert, South Africa. We found that dominant females, the core member in meerkat social systems, led groups to the road significantly more often than subordinates, yet were consistently less likely to cross first. Our results suggest that a reshuffling occurs in progression order when meerkat groups reach the road. By employing a simple model of collective movement, we have shown that risk aversion alone may be sufficient to explain this reshuffling, but that the risk aversion of dominant females toward road crossing is significantly higher than that of subordinates. It seems that by not crossing first, dominant females avoid occupying the most risky, exposed locations, such as at the front of the group – a potential selfish strategy that also promotes the long-term stability and hence reproductive output of their family groups. We argue that our findings support the idea that animals can flexibly apply phylogenetically-old behavioural strategies to deal with emerging modern-day problems.

## Introduction

When faced with a heightened risk of predation during group movements, animals often display adaptive spatial patterning to minimize danger. Sand fiddler crabs (*Uca pugilator*) and redshanks (*Tringa totanus*), for example, reduce the distance to their nearest group neighbour such that the whole flock becomes more cohesive during predation events [1], [2]. Such spatial re-orientation is thought to diminish relative predation risk by reducing an individual’s Domain of Danger – the area in which an animal is vulnerable to predation [3]. Given that Domain of Danger reduction always occurs at the expense of peripheral, subordinate group members, it has previously been referred to as “selfish herding” behaviour [3]. In contrast, animals can also spatially position themselves to reduce the danger experienced by other group members. Dominant male baboons (*Papio cynocephalus*), for example, occupy exposed socio-spatial positions, particularly the front and rear of the group, when moving through known risky areas [4]. Such a strategy is instead thought to serve a protective function, minimizing the risk experienced by more vulnerable group members [5].

With the continual encroachment of humans into the natural habitat of animals, not only ecological threats, but also man-made challenges, represent a considerable emerging danger. Over the last century, roads in particular have fragmented the habitats of a huge range of species, disturbing the natural movement of animals and ultimately hindering foraging behavior and potential mate finding [6]. Exactly how animals respond to such recent human-imposed threats is, however, surprisingly less clear [7].

Meerkats (*Suricata suricatta*), cooperatively breeding social mongoose, are exposed to a range of external ecological threats, including both terrestrial and aerial predators [8]. In recent times, human-induced threats, especially the encroachment of roads, have also begun to play an increasingly large role in the day-to-day lives of meerkats. We studied progression order and spatial disturbance in response to a single large and dangerous road in meerkat groups living in the Kalahari Desert, South Africa. For the groups whose home range is dissected by the road, traffic contributes substantially to overall mortality rates (Kalahari Meerkat Project, unpublished data). We therefore hypothesized that despite the relatively recent presence of the road, meerkats would draw on adaptive socio-spatial patterning that evolved in naturalistic risky contexts, to manage the dangers associated with encountering this unnatural obstacle within their territory. In line with previous studies on risk aversion in animals we focused primarily on the relative spatial position of the core dominant individual, which in the case of meerkat social systems, is the breeding female [9]. Given the importance of this key individual in maintaining the stability of the social group, we expected that dominant females would demonstrate greater risk aversion in response to the road than subordinates. Specifically, we predicted dominant females would minimise their relative risk through occupying more “protected” spatial positions during road-crossing, driven by a delay in tendency to cross the road first. We used a minimalistic model of collective movement [10] to quantitatively capture the extent of this risk-averse behaviour, depending on an individual's dominance status.

## Methods

### Study Site and Subjects

**Figure 1:**
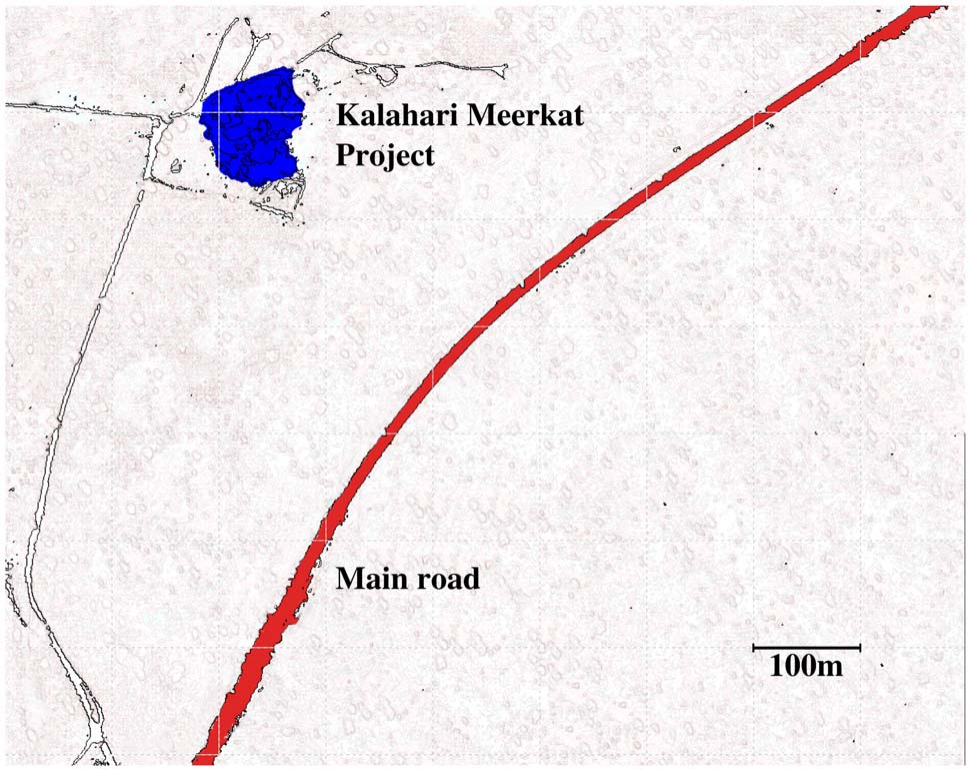
Ground map of the Kuruman River Reserve (North is up) indicating the Kalahari Meerkat Project (blue area) and the main road running NE-SW (red).

Observations of road crossings were conducted on 4 groups of wild, but habituated, meerkats at the Kalahari Meerkat Project (KMP), Kuruman River Reserve, South Africa [11]. Data was collected ad libitum during morning and evening observation sessions between September 2009 and July 2010. Road-crossing observations were restricted to a single main road running parallel to the Kuruman River Reserve (see Fig. 1). This road is particularly busy due to the high volumes of traffic travelling between popular tourist destinations within the Northern Cape. As part of the KMP`s long-term data collection, all animals were tagged with sub-cutaneous transponders and with dye markings for individual identification [9]. All subjects were habituated to a level that allowed close behavioural observations within 1 m.

### Observational Data Collection

Road crossing was defined as when a complete group of meerkats terminated foraging, approached the road together and traversed it in a continuous, fluid motion. To avoid ambiguity in identifying progression order we did not consider events where meerkats approached the road together but then, due to ongoing external events, such as inter-group conflicts or predation events, split into subgroups, which then crossed independently.

During road crossing events we recorded which individuals led the group (classed as the individual at the front) towards the direction of the road following termination of foraging. We specifically recorded if the individuals were dominant (one of the breeding pair) or subordinate and, when negotiating the road, the subsequent position of the leading individual, particularly if they remained at the front of the travelling group. To rule out the possibility that previous experience modulates risk sensitivity to the road, we compared the ages of subordinate individuals leading and crossing first with those who also led but did not cross first.

### Statistics

Owing to the non-normal nature of the data, we employed non-parametric statistical tests. We used proportion tests to analyse the effect of dominance status on the likelihood of an individual leading the group to the road. The number of road-crossings led by subordinates and dominants was divided by the total number of dominant females and subordinate individuals involved in each road-crossing event allowing us to control for the fact that the relative proportion of dominant females to subordinates within a group was highly skewed. We then used binomial tests to investigate how dominant females and subordinates differed in their probability to cross the road first if they were leading. To calculate the expected level of chance we first calculated the frequency of crossing first given prior leading for each individual, and divided the sum by the total number of individuals. Exact Mann-Whitney U tests [12] were used to analyse the effect of age on the tendency to avoid crossing the road first when initially leading. All tests were two tailed and implemented in SPSS (v.19.0) and R (v. 2.12). Alpha values were set at 0.05.

### Self-propelled Particle Model

To determine the magnitude of the perturbation that the group undergoes when reaching the road, we implemented the crossing situation in a self-propelled particle (SPP) model with a drift component towards the road and a deflecting barrier at the edge of the road. We used the general SPP model proposed by Vicsek et al. [13], which has been extensively studied in the context of collective motion and is arguably the *de facto* standard for minimalistic models of animal groups on the move [see 9, Chap. 5; 14]. The discrete expression of the motion in two dimensions reads:
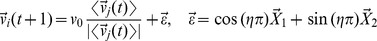


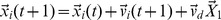
where X_1_ and X_2_ are the base vectors of the space (X_1_ points towards the road and X_2_ is perpendicular to it), and x_i_ and v_i_ are the position and velocity vectors of individual i, respectively. v_0_ and v_d_ are the constant norms of the coherent movement and the drift component towards the road, respectively. The unit vector which multiplies v_0_ is the average direction of motion of all the individuals, and ε controls for the amount of stochastic noise in the system (η is uniformly distributed in [-η_max_,η_max_]). We assume a fully-cohesive regime in which all individuals adapt their trajectory to one another's, so that the group does not split before reaching the road. In the simulations, we used η_max_ = 0.3, which is around the threshold for ordered motion [13], and v_d_ = v_0_/2, so that the collective dynamics of the movement are stronger than the drift component, but the group still reaches the road in a reasonable time.

When the group reaches the road (a line at X_1_ = X_r_ parallel to X_2_), the obstacle acts as a potential barrier by deflecting the particles whose energy is not high enough to cross it; in other terms, we assume an elastic collision between the immobile barrier and the moving particle. If Θ(t) is the direction of motion at time t, its forward component is F = v_0_cos(Θ(t))+v_d_. The particle hits the barrier if F>X_r_-X_1_(t). With a barrier of height H, there are two possible cases:
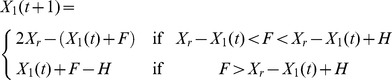



The first case corresponds to the particle bouncing against the barrier, the second to the particle effectively crossing the barrier. For simplicity, we assume that the normal motion (parallel to X_2_) is unaffected by the barrier, which does not change the results.

## Results

**Figure 2:**
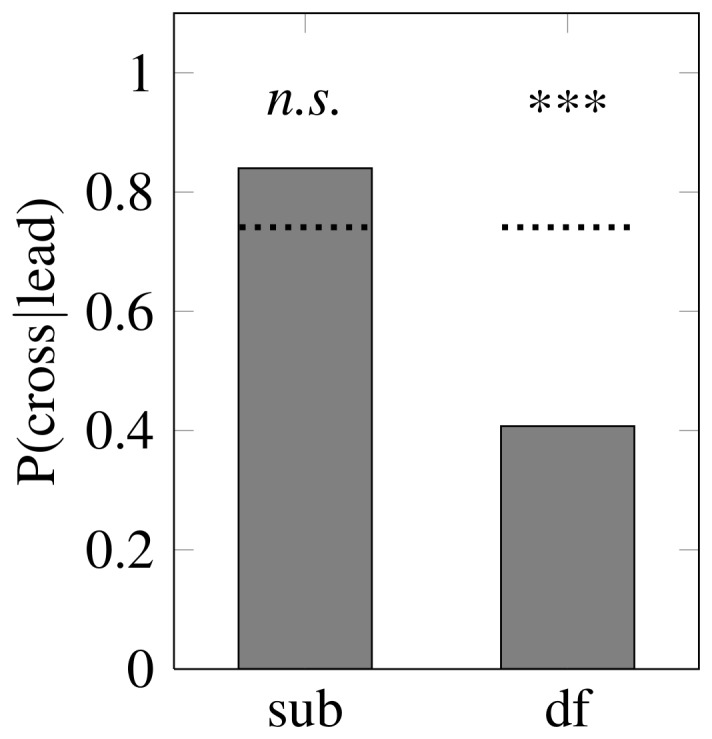
Probability of crossing the road first given prior leading, for both subordinate individuals and dominant females (*** = p<0.001).

We recorded a total of 52 road-crossing events. Although dominant female meerkats contributed less to the overall group size in comparison to subordinates, they were significantly more likely to lead the group to the road (mean proportion of dominant females leading = 0.52, mean proportion of subordinates leading = 0.48, 2-tailed Proportion test with continuity correction, χ^2^ = 63.0, df = 1, p<0.001, see Fig. 2). On some occasions the dominant male was observed travelling towards the front of the group (pers. obsv.), however we never observed him to lead the group towards the road.

Despite leading more, dominant females were significantly less likely to remain at the front of the group and cross first than subordinates: we observed that the dominant female crossed the road given her prior leading in 11 out of 27 instances –41% –, whilst this was the case for subordinates in 21 out of 25 instances –84% – (2-tailed binomial test for dominant females, test proportion = 0.741, p<0.001, 2-tailed binomial test for subordinate individuals, test proportion = 0.741, p = 0.984, see Fig. 2). We found no significant difference between the age of subordinates crossing first (mean age of subordinate crossing first = 14.9 months, mean age of subordinate not crossing first = 18.2 months, Exact Mann-Whitney U test, Z = −1.42, p = 0.168).

**Figure 3:**
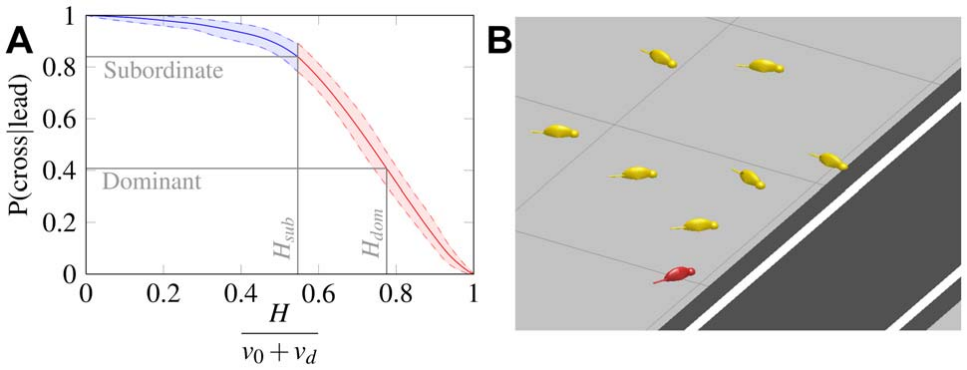
a. Model results: the probability of an individual crossing the road first given prior lead is given as a function of the perceived barrier height, or level of risk aversion. H_sub_ and H_dom_ are the relative barrier heights for subordinate individuals and dominant females, respectively. Shaded (and dashed) areas mark the 95% confidence interval of the probability estimate. b. Illustration of a group of 8 meerkats negotiating the road. The leader (first individual to cross the road) is coloured in red. Note that the behaviour of the leader is only different to that of the other individuals during the crossing phase, not during the approach (foraging) phase. The corresponding simulation of the self-propelled particle model is provided as an animated visualisation sequence in the supplementary information.

The model allowed us to quantify the extent of the reshuffling occurring at the front of the group upon reaching the road. We ran simulations (100'000 for each value of the barrier height) of road crossing in groups containing the average observed number of individuals (n = 8) and with increasing barrier heights (with a null barrier, the road induces no reshuffling in progression order). This allowed us to capture the level of risk aversion that may be responsible for the observed reshuffling when a subordinate is leading. Because in our model the height of a crossable barrier can only lie between 0 and v_0_+v_d_ (above which the obstacle is too great to be crossed in one time step and the particles bounce endlessly against it), we can conveniently express this height as a fraction of v_0_+v_d_ (see Fig. 3a). We found that the height corresponding to the perturbation in progression order among dominants and subordinates was H_sub_ = 0.55/(v_0_+v_d_) (95% confidence interval: 0.50–0.59). We then ran simulations in which the barrier may have a different height for the leading individual (the first individual to reach the road) than for the others, in order to express a potentially different level of risk perceived. We found that the height corresponding to the amount of reshuffling observed in the cases where the dominant female is leading was significantly higher, at H_dom_ = 0.78/(v_0_+v_d_) (95% CI: 0.74–0.81). This translates to a risk aversion towards the road about 42% higher for the dominant female than for the subordinate individuals. These results are illustrated in Fig. 3a, and a video of the model is provided in the supplementary information (see also Fig. 3b).

## Discussion

When exposed to dangerous ecological situations during group movement, animals demonstrate adaptive risk-averse behaviour. Whether they also employ similar fitness-enhancing strategies when faced with more recent man-made threats is particularly intriguing, primarily because it indicates a capacity for flexible problem solving through applying phylogenetically-old behavioural strategies [7]. We found that meerkats do indeed demonstrate risk-averse behaviour in response to a large man-made road running through their territory. Observational data suggests that although dominant females are more likely to lead the group towards the road, upon reaching this artificial obstacle a reshuffling in progression order occurs. In comparison to subordinate individuals who will often cross the road first if they are leading, dominant females seemingly abandon the leading edge, instead dropping back into the core of the crossing group. To make a qualitative assessment of the level of this risk aversion, we needed a simple yet accurate assumption as to how a foraging group may move towards the road as a collective, and how individuals react to it. By adapting a well-known, yet minimalistic, model of collective motion to this scenario, we have been able to show that risk aversion in the form of repulsion towards the road may be sufficient to explain the changes in progression order, both when a subordinate individual and the dominant female are leading the group to the road. Under the assumptions we made (cohesive group movement with a drift component towards the road), we have found that our observations translate into a level of risk aversion over 40% higher for the dominant female than for subordinates.

Our results support recent findings demonstrating similar risk sensitivity in response to man-made roads in wild chimpanzees (*Pan troglodytes*) [7]. In both instances dominant individuals displayed risk-aware behaviour, however dramatic differences exist in the exact behavioural strategy employed during road-crossing. In chimpanzees dominant males assumed protective socio-spatial positions, either at the front or the rear of the group, whereas dominant meerkat females seemed to rather minimize their own relative risk by actively avoiding such exposed positions. In contrast to chimpanzees, the dominant female and not the dominant male is considered the core individual in meerkat social groups [9]. In fact, in some instances, when a dominant female is predated, meerkat groups have been observed to destabilise and sometimes break down into dispersing individuals (KMP unpublished data). This increased risk aversion experienced by the dominant female could simply represent a byproduct of social dominance; subordinates are constrained to cross first. However, it may also be that this selfish, risk-aware behaviour of dominant females at the proximate level functions adaptively, reducing their probability of injury or death on the road and simultaneously enhancing group stability and long-term reproductive output of herself and her kin group members.

This avoidance strategy nicely mirrors previous theoretical and empirical “selfish herding” findings demonstrating that dominants reduce their domain of danger at the expense of subordinates [1], [3]. Unfortunately it was not possible to accurately measure the distance between the dominant female and subordinates during road-crossing and hence through which mechanisms meerkats vary their socio-spatial positions at the more subtle level to reduce, for example their domain of danger, remains unclear. Future work, however, aims to more accurately capture the individual and collective dynamics of meerkat movement and behaviour in response to both natural and man-made exogenous perturbations. This work will also be beneficial in helping to further clarify the exact adaptive nature of the behavioural differences observed.

With the size of the human population on Earth now exceeding seven billion [15], the likelihood of human and animal habitats colliding is only going to intensify. We show, however, that when meerkats are faced with a recent human-induced dangerous obstacle they can flexibly adapt phylogenetically-old risk-sensitive behaviours, to process and react to novel threats. These results provide a glimmer of hope for the notion that animals can adapt and co-exist successfully with humans, despite our ever-increasing encroachment. We hope our findings will encourage further work into this newly-emerging field of animal cognition where the problems which need solving are not only within the natural environment of animals but are also those brought about by human presence.

## Supporting Information

Video S1 **Video of a simulation of the self-propelled particle model; this animation represents a group of 8 meerkats negotiating the road.** The leader (first individual to cross the road) is coloured in red. Note that the behaviour of the leader is only different to that of the other individuals during the crossing phase, not during the approach (foraging) phase.
(MP4)
